# Comparison and Noise Suppression of the Transmitted and Reflected Photoplethysmography Signals

**DOI:** 10.1155/2018/4523593

**Published:** 2018-09-26

**Authors:** Suyi Li, Lijia Liu, Jiang Wu, Bingyi Tang, Dongsheng Li

**Affiliations:** ^1^College of Instrumentation and Electrical Engineering, Jilin University, Changchun, China; ^2^National Geophysical Exploration Equipment Engineering Research Center, Jilin University, Changchun, China

## Abstract

The photoplethysmography (PPG) is inevitably corrupted by many kinds of noise no matter whether its acquisition mode is transmittance or reflectance. To enhance the quality of PPG signals, many studies have made great progress in PPG denoising by adding extra sensors and developing complex algorithms. Considering the reasonable cost, compact size, and real-time and easy implementation, this study proposed a simple real-time denoising method based on double median filters which can be integrated in microcontroller of commercial or portable pulse oximeters without adding extra hardware. First, we used the boundary extension to preserve the signal boundary distortion and designed a first median filter with the time window at approximately 78 ms to eliminate the high-frequency components of the signal. Then, through the second median filter with a time window which was about 780 ms, we estimated the low-frequency components. Finally, we removed the estimated low-frequency components from the signal to obtain the denoised signal. Through comparing the multiple sets of signals under calmly sitting and slightly moving postures, the PPG signals contained noises no matter whether collected by the transmittance-mode or the reflectance-mode. To evaluate the proposed method, we conducted measured, simulated experiments and a strong noisy environment experiment. Through comparing the morphology distortions, frequency spectra, and the signal-to-noise ratios (SNRs), the results showed that the proposed method can suppress noise effectively and preserve the essential morphological features from PPG signals. As a result, the proposed method can enhance the quality of PPG signals and, thus, can contribute to the improvement of the calculation accuracy of the subsequent physiological parameters. In addition, the proposed method could be a good choice to address the real-time noise reduction of portable PPG measuring instruments.

## 1. Introduction

The initial clinical application of photoplethysmography (PPG) is to monitor the noninvasive blood oxygen saturation (SpO_2_) and, furthermore, it has been widely used in the assessment of the cardiovascular, respiratory, and hematological status [1, 2].

The principle of PPG acquisition is to emit different wavelengths of LED lights on the epidermis and to receive the transmitted or reflected lights by using the photoelectric sensors. The received optical density will vary as the blood volume of the measuring site changes and can be recorded by using electrical signals to form PPG which can be used to derive the approximate formula for SpO_2_ estimation based on Lambert–Beer law [3, 4]. Compared with the fingertip transmittance-mode PPG monitoring, the measuring site and the motion status are relatively flexible under the reflectance-mode, and studies have shown that the reflectance-mode way can achieve better SpO_2_ measurement accuracy during the perfusion [5, 6]. However, finger-tip transmittance pulse oximeters are still playing the important role in clinical applications because of their stable performance, easy operation, and low cost [7].

No matter whether the PPG signal is obtained by using transmittance-mode or reflectance-mode, it will inevitably be corrupted by many kinds of noise such as high-frequency noise, power line interference, baseline drift, and motion artifact (MA), and these noises will affect the pulse rate analysis and SpO_2_ measurement accuracy [8]. To date, many studies have proposed noise suppression methods for PPG signals. The high-frequency noise in PPG signal could be eliminated by empirical mode decomposition (EMD) method [9]. The power line interference could be reduced by a wavelet denoising method integrated in DSP [10]. The baseline drift could be estimated by applying the wavelet multiresolution principle [11, 12]. A real-time method based on a contour analysis was implemented on a 32-bit ARM core microcontroller to detect the pulse waveform segmentation and artifact [13]. Due to the fact that MA can significantly distort the morphology of the PPG signal, it is worthwhile to focus on the removal methods. One of the commonly used approaches is through adding the extra hardware, such as using the accelerometer as the reference signal for MA cancellation [14–16]. Another way is through designing the denoising algorithms, including adaptive filter [17, 18], wavelet-based method [19, 20], independent component analysis (ICA) [21, 22], singular value decomposition (SVD) [23], cycle-by-cycle Fourier series analysis [24, 25], and higher order statistics [26].

We have seen the significant progress in the domain of PPG denoising. However, the above methods include adding extra hardware and requiring high-end microcontroller or host computer where the complicated algorithm needs to run and the PPG data needs to be transferred to, which are all complicated and can be inapplicable due to the high market share of the traditional pulse oximeter. Therefore, considering the low cost, compact size, and real-time and easy implementation, a denoising method based on double median filters is proposed in this study. The proposed denoising method can run on the ordinary microcontroller and can be real-time. In other words, it is convenient enough to be embedded in commercial two-wavelength pulse oximeters without changing any hardware or transmitting the data to the host computer for processing.

This study will first compare the reflected PPG with the transmitted PPG signals under calmly sitting and slightly moving postures and describe the principle of the denoising method based on double median filters and its implementation steps. Then, we will conduct experiments using measured signals, simulated signals, and the noisy signals collected under the strong noise environment, respectively. Finally, we will evaluate the performance of the proposed denoising method through comparing the morphology distortions, frequency spectra, and SNRs.

## 2. Materials and Methods

### 2.1. The Measurement Devices

**Figure 1:**
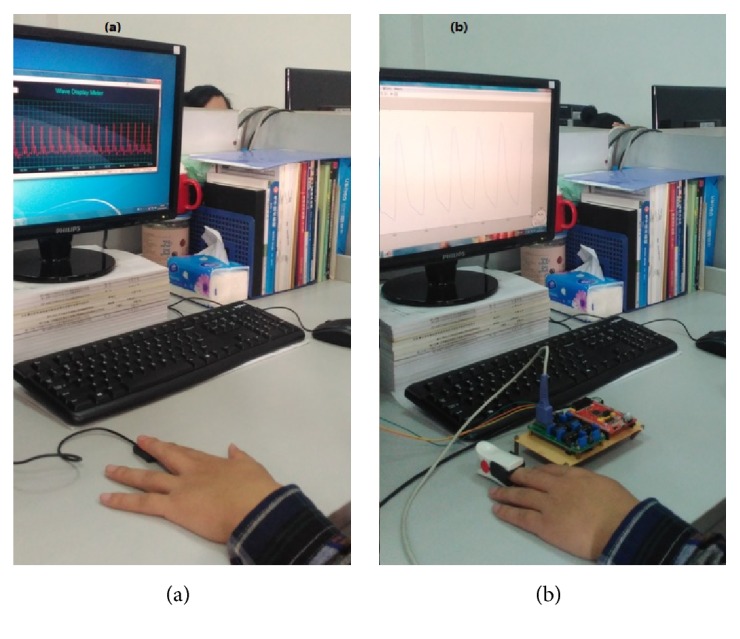
Pulse oximeters: (a) reflectance-mode, (b) transmittance-mode.

Most pulse oximeters are designed based on the good linear relationship between the oxygen saturation and the relative light intensity of the 660 nm (red-light) and 940 nm (IR-light) wavelengths received by the photodetector. The reflective pulse oximeter used in the experiment was provided by Tianjin Synopsis Technology Co., Ltd., China. The raw data can be collected by the data acquisition software provided by the company with the sampling frequency of 100 Hz. The emitter and photodetector are adjacent to each other with the measuring site side by side, as shown in Figure 1(a). The prototype of transmitted pulse oximeter was developed by Jilin University, China, with the sampling frequency of 128 Hz. The emitter and photodetector are opposite to each other with the measuring site in-between, as shown in Figure 1(b). When the red-light and IR-light pass through the measuring site, they will be received by the photodetector to produce PPG signals, as shown in Figure 1.

### 2.2. PPG Signals Acquisition

A total of 10 volunteers participated in the experiment, 7 males and 3 females. The mean age (mean ± std) was 26.20 ± 5.14 and the mean body mass index (BMI ± std) was 21.79 ± 3.40. The volunteers were informed about the study before the data was obtained.  Table 1 shows their basic personal information.

We used the above two devices to collect their multiple sets of middle fingertips PPG signals under the calmly sitting and slightly moving postures. Hardware filters can eliminate some of the noise in PPG signals, but the signal was still affected by respiration, random noise, and motion artifacts during the measuring procedure, resulting in morphological distortions in PPG signals. For the clarity of the subsequent comparisons, the amplitude range of the signal was normalized from 0 to 1 by using

(1) $$\begin{array}{c} PPG1\left(\;i\;\right)=\frac{PPG\left(i\right)-min\left(PPG\left(i\right)\right)}{max\left(PPG\left(i\right)\right)-min\left(PPG\left(i\right)\right)} \end{array}$$

where *i* = 1,2,…, *L*, *L* is the data length, *PPG*1 is the normalized signal, and *PPG* is the raw signal.

**Figure 2:**
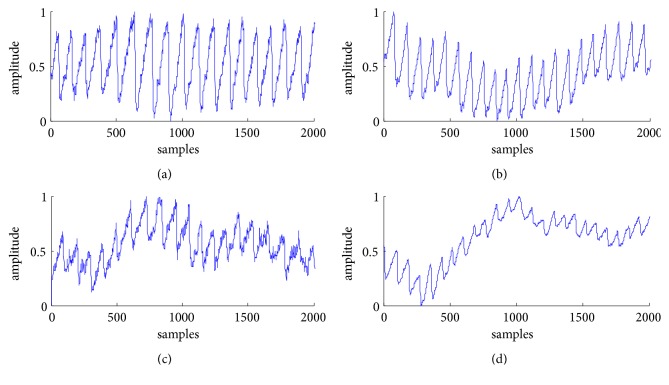
PPG signals: (a) the transmittance-mode in sitting calmly, (b) the reflectance-mode in sitting calmly, (c) the transmittance-mode in moving slightly, and (d) the reflectance-mode in moving slightly.

We employed 2000 representative samples which were taken from the IR signals acquired by the transmittance and by the reflectance oximeter, respectively, as shown in Figure 2. The quality of the PPG signal under the sitting posture is relatively good.  Figure 2(a) illustrates the transmitted signal which contained a small amount of high-frequency noise; Figure 2(b) shows the reflected PPG signal with mild baseline wander which may be caused by respiration. Under the slightly moving posture, the morphologies of the transmitted and reflected signal are both distorted due to the motion artifacts, as shown in Figures 2(c) and 2(d), respectively. Therefore, it is necessary to suppress the noise and to preserve the essential morphological features, enhancing the signal quality to improve the calculation accuracy of the physiological parameters subsequently.

### 2.3. The Denoising Method Based on Double Median Filters

According to the frequency of the major component of the PPG signal and the spectral comparisons of the reflected and the transmitted signals under the calmly sitting and slightly moving postures, we designed a noise reduction method based on double median filters.

The median filter is a nonlinear digital filter technique which is very widely used to eliminate noise from digital signals. It works by moving through the signal entry by entry, replacing each entry with the median of neighboring entries. The pattern of neighbors is called the “window,” which slides, entry by entry, over the entire signal [27]. The window size is the key step of the median filter design. In general, the larger the window is, the lower the frequency of the fitted signal is.

Through the simulation and measurement experiments, the high-frequency noise can be suppressed effectively when the window size is from 9 to 12 samples under the sampling frequency of 128 Hz; the low-frequency noise introduced mainly by respiration and movement can be reduced better when the window size is from 96 to 110.

**Figure 3:**
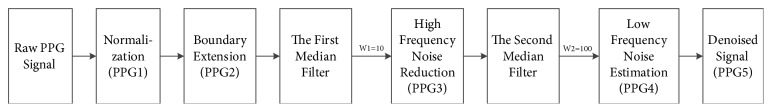
Block diagram of the denoising method based on double median filters.

In this application, the window size of the first median filter, *W*1, was set as 10 samples, corresponding approximately to 78 ms; that of the second median filter, *W*2, was set as 100 samples, corresponding approximately to 780 ms. The diagram of the denoising method is shown in Figure 3.

The main steps of the method are shown as follows.

(1) The raw signal after normalization is denoted as *PPG*1. In order to prevent the boundary distortion, we used the boundary extension to process *PPG*1. The extension length *Q* is related to the size of the window; here we chose *Q* = *W*2/2 = 50, and the signal after extension is recorded as *PPG*2, shown in

(2) $$\begin{array}{c} PPG2\left(\;n\;\right)=\left\{\begin{array}{cc} PPG1\left(1\right), & 1\leq n\leq 50\\ PPG1\left(n-50\right), & 50<n\leq 2050\\ PPG1\left(2000\right), & 2050<n\leq 2100 \end{array}\right. \end{array}$$

(2) The first median filter with the window size *W*1 = 10 was used to eliminate the high-frequency noise from *PPG*2; then the signal after the processing is recorded as *PPG*3, and the value of *PPG*3(*n*) equals the one from the sequence *PPG*2(*i*) whose value corresponds to the minimum of the expression ∑_*i*=*n*−5_^*n*+4^|*PPG*2(*i*) − *PPG*3(*n*)|, where 51 ≤ *n* ≤ 2050, *n* − 5 ≤ *i* ≤ *n* + 4.

(3) The second median filter with the window size *W*2 = 100 could be used to estimate the low-frequency noise of *PPG*3 which is recorded as *PPG*4; then the value of *PPG*4(*n*) equals the one from the sequence *PPG*3(*i*) whose value corresponds to the minimum of the expression ∑_*i*=*n*−50_^*n*+49^|*PPG*3(*i*) − *PPG*4(*n*)|, where 51 ≤ *n* ≤ 2050, *n* − 50 ≤ *i* ≤ *n* + 49.

(4) The denoised signal *PPG*5 can be obtained by subtracting *PPG*4 from *PPG*3, as shown in

(3) $$\begin{array}{c} PPG5\left(\;n\;\right)=PPG3\left(\;n\;\right)-PPG4\left(\;n\;\right),\;51\leq n\leq 2050 \end{array}$$

## 3. Results and Discussion

### 3.1. The Experiment for Measured Signals

In the measurement experiment, the above two devices were used to collect multiple sets of transmitted and reflected PPG signals, of which the 10 subjects were under the calmly sitting and slightly moving postures. For convenient comparison, we continued to use the signals in Figure 2 to represent the denoising results.

**Figure 4:**
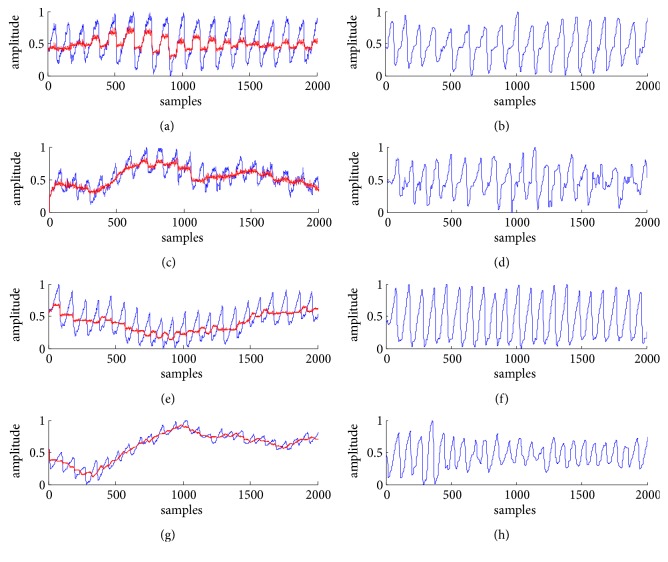
The denoising results by using the method based on double median filters: (a) TPPGS and the noise (red curve), (b) DTPPGS, (c) TPPGM and the noise (red curve), (d) DTPPGM, (e) RPPGS and the noise (red curve), (f) DRPPGS, (g) RPPGM and the noise (red curve), and (h) DRPPGM.

The red curves in Figure 4 are the noise suppressed by using our proposed method.  Figure 4(a) is the raw transmitted PPG signal (blue curve) under the sitting posture, named as TPPGS, and Figure 4(b) is that under the slightly moving posture, named as TPPGM; their corresponding denoised signals are shown, respectively, in Figure 4(c), named as DTPPGS, and in Figure 4(d), named as DTPPGM.

Figure 4(e) is the raw reflected PPG signal (blue curve) under the sitting posture, named as RPPGS, and Figure 4(f) is that under the slightly moving posture, named as RPPGM; their corresponding denoised signals are shown in Figure 4(g), named as DRPPGS, and in Figure 4(h), named as DRPPGM.

From visual comparison of the morphology and the smoothness of the signals before and after denoising, the noise is well suppressed, and the quality of the PPG signals is improved by the denoising method.

**Figure 5:**
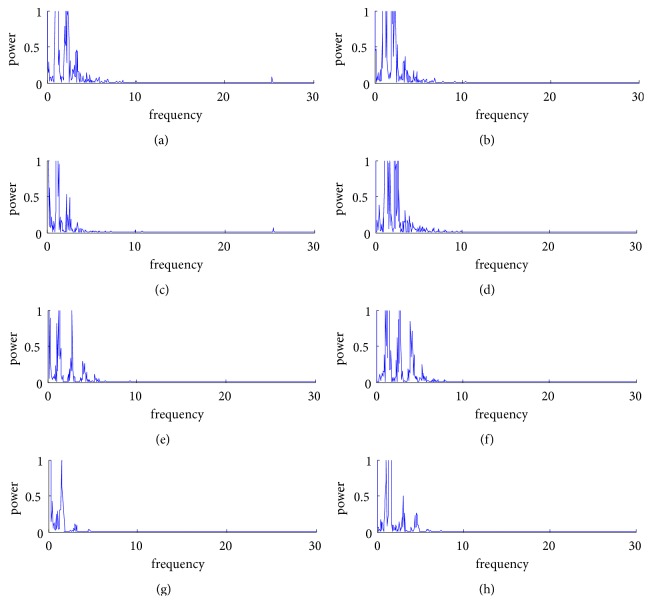
Comparisons of spectral analysis before and after denoising: (a) spectrum of TPPGS, (b) spectrum of DTPPGS, (c) spectrum of TPPGM, (d) spectrum of DTPPGM, (e) spectrum of RPPGS, (f) spectrum of DRPPGS, (g) spectrum of RPPGM, and (h) spectrum of DRPPGM.

For quantitative evaluation of the method, we performed spectral analysis of the PPG signals from Figure 4. The frequency of the major component of PPG signal is generally concentrated in 0.5-10 Hz; hence, the frequency values plotted in Figure 5 are set from 0 to 30 Hz for convenient observation. Figures 5(a)–5(h) correspond to the spectra of TPPGS, of DTPPGS, of TPPGM, of DTPPGM, of RPPGS, of DRPPGS, of RPPGM, and of DRPPGM, respectively. It can be found that the high frequency near 25 Hz and the low frequency near 0.5 Hz are well eliminated.

### 3.2. The Experiment for Simulated Signals

We compared the denoising effects of the proposed method and a wavelet-based method. The wavelet-based method can remove baseline wander as well as partial motion artifacts effectively. However, it should be run on the host computer and it is not real-time. We used a fraction of good quality signals to be the reference signal. The noisy signals could be synthesized by adding simulated noise to the reference signal. Then, the two denoising methods were compared by using SNR.

(4) $$\begin{array}{c} SNR=20\;\log_{10}\left(\;\overset{N}{\underset{n=1}{\sum }}\frac{s{\left(n\right)}^{2}}{{\left|x\left(n\right)-s\left(n\right)\right|}^{2}}\;\right). \end{array}$$

**Figure 6:**
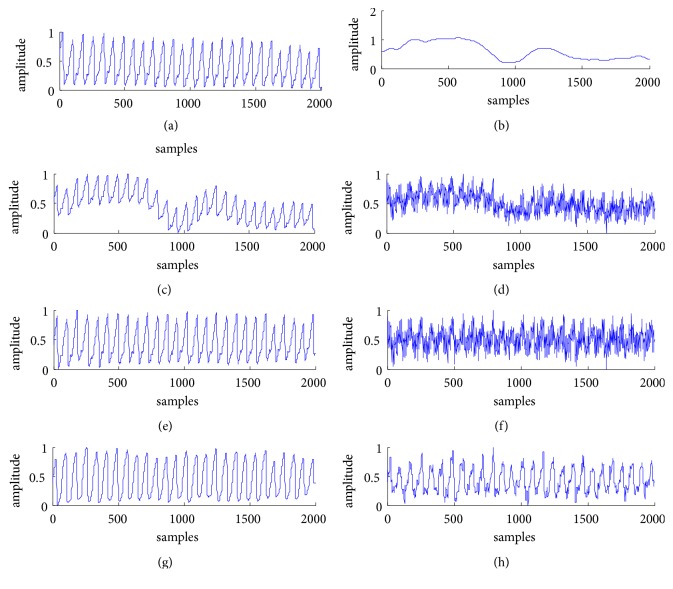
Comparisons of the proposed method with the wavelet-based method.** (a**) The reference signal, (**b**) the simulated noise for baseline wander and partial motion artifacts, (**c**) the simulated noisy signal with baseline wander and partial motion noise, (**d**) the simulated noisy signal with multinoise, (**e**) the denoised signal of Figure 6(c) by using the wavelet-based method, (**f)** the denoised signal of Figure 6(d) by using the wavelet-based method, (**g**) the denoised signal of Figure 6(c) by using the proposed method, and (**h**) the denoised signal of Figure 6(d) by using the wavelet-based method.

We used 2000 representative samples from good quality reflected signals to be the reference signal, as shown in Figure 6(a). The simulated noise for baseline wander and partial motion artifacts is shown in Figure 6(b). The simulated noise being superposed on the reference signal synthesized a noisy signal, which is shown in Figure 6(c). We continue to add 10 dB white Gaussian noise to synthesize another noisy signal which contains not only the baseline wander and partial motion artifacts, but also the random noise, as shown in Figure 6(d). After using the wavelet-based method (please see [11, 12] for details), the denoised signal of Figure 6(c) is shown in Figure 6(e), and that of Figure 6(d) is shown in Figure 6(f). After using our proposed method, the denoised signal of Figure 6(c) is shown in Figure 6(g), and that of Figure 6(d) is shown in Figure 6(h).

The corresponding SNRs of the signals from Figure 6 were calculated. The comparison results are listed in Table 2.

The SNR calculated by using the noisy signal in Figure 6(c) is 7.6857 and those using the denoised signals in Figures 6(e) and 6(g) are 16.2620 and 12.7598, respectively; the SNR calculated by using the noisy signal in Figure 6(d) is 5.7739 and those using the denoised signals in Figures 6(f) and 6(h) are 6.1874 and 11.3860, respectively.

By comparing the morphology in Figure 6 and the SNR in Table 2, we can see that the quality of the signal is improved after denoising. Comparing Figures 6(c), 6(e), and 6(g), the noise is reduced well. Although the denoised effect by the wavelet-based method (SNR is 16.2620) is better than that by the proposed method (SNR is 12.7598), the wavelet-based method needs to run on the host computer and is not real-time. Comparing Figures 6(d), 6(f), and 6(h), the wavelet-based method is good at reducing that kind of low-frequency noise, but not the random noise, and the denoised effect of the proposed method (SNR is 11.3860) is much better than that by the wavelet-based method (SNR is 6.1874).

### 3.3. The Experiment for Strong Noisy Signals

**Figure 7:**
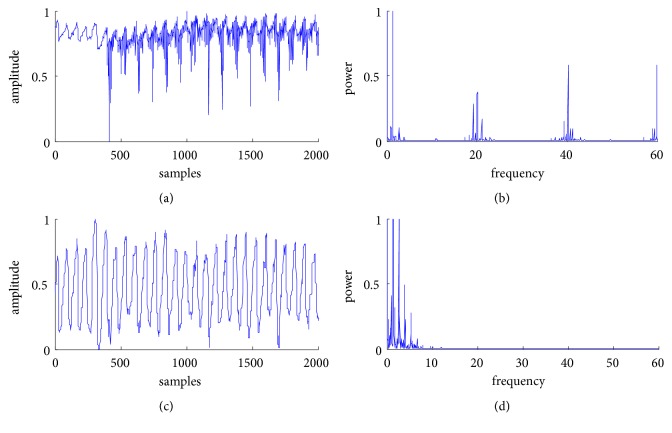
Spectral analysis of the reflected PPG signal before and after denoising under strong noise background: (a) the raw PPG signal, (b) the spectrum of the raw signal, (c) the denoised PPG signal, and (d) the spectrum of the denoised signal.

During the experiment, it happened to start a suction electromagnetic vibrator that greatly increased the environmental noise in the measurement. At that time, we collected the raw signal by using the reflective oximeter. The representative 2000 samples are shown in Figure 7(a).  Figure 7(b) is the spectrum of the raw signal, and the noise is severe in the signal.  Figure 7(c) is the denoised signal using the method based on double median filters, and Figure 7(d) is the spectrum of the denoised signal. By comparing the morphology and spectrum of the signal before and after denoising, the validity of the denoising method is further verified.

The above denoising experiments used measured signals, simulated signals, and the strong noisy signals. By comparing the morphology, frequency spectrum, and SNR of the signals before and after denoising, the results demonstrated the effectiveness and practicability of the proposed method.

## 4. Conclusion

In this study, a simple, real-time PPG denoising method based on double median filters was proposed, which could be integrated in microcontroller of commercial or portable pulse oximeters without adding extra hardware. The experiments were conducted using measured signals, simulated signals, and the noisy signals collected under the strong noise environment. Through evaluating the denoising effects (morphology, frequency spectrum, and SNR comparisons of the signals), the experimental results showed that the proposed method can remove the noise well and enhance the quality of PPG signals. The proposed method has the potential to improve the calculation accuracy of the subsequent physiological parameters and can be a convenient solution to the real-time noise suppression for portable pulse oximeters as well. For future research, we will collect more PPG signals from volunteers to compare the noise reduction effects through the calculation accuracies of physiological parameters (such as HR and SpO_2_) before and after denoising using the proposed method and traditional methods. And then, results will further reveal the feasibility and possibility of embedding the proposed method into commercial portable pulse oximeters.

## Figures and Tables

**Table 1:** The basic personal information of subjects who participated in the experiment.

Subject	Gender	Age (year)	Height (cm)	Weight (kg)	BMI
1	male	24	184	85	25.1
2	female	22	160	44	17.2
3	male	25	170	75	26.0
4	male	23	175	75	24.5
5	female	23	162	48	18.3
6	male	39	170	62	21.5
7	male	31	178	75	23.7
8	female	25	158	45	18.0
9	male	24	173	74	24.7
10	male	26	178	60	18.9

**Table 2:** The comparison results of SNRs.

Noisy signals	Denoised signals	
	by using the wavelet-based method	by using the proposed method
7.6857	16.2620	12.7598
5.7739	6.1874	11.3860

## Data Availability

The data used to support the findings of this study are available from the corresponding author upon request.

## Abbreviations

PPG: Photoplethysmography
SpO_2_: Noninvasive blood oxygen saturation
MA: Motion artifact
EMD: Empirical mode decomposition
ICA: Independent component analysis
SVD: Singular value decomposition
BMI: Body mass index
SNR: Signal-to-noise ratio
TPPGS: Transmitted PPG signal
DTPPGS: Denoised transmitted PPG signal
TPPGM: Transmitted PPG signal under moving
DTPPGM: Denoised transmitted PPG signal under moving
RPPGS: Reflected PPG signal
DRPPGS: Denoised reflected PPG signal
RPPGM: Reflected PPG signal under moving
DRPPGM: Denoised reflected PPG signal under moving.
